# Progression to microalbuminuria in patients with type 1 diabetes: a seven-year prospective study

**DOI:** 10.1186/1758-5996-3-21

**Published:** 2011-08-26

**Authors:** Roberta A Cobas, Bráulio Santos, Pedro CB da Silva, Ricardo Neves, Marilia B Gomes

**Affiliations:** 1 Division of Diabetes, Department of Medicine, State University of Rio de Janeiro, Rio de Janeiro, Brazil; 2 Biostatistics and Bioinformatics Unit, National Institute of Cardiology, Rio de Janeiro, Brazil; 3 Division of Ophthalmology, Department of Medicine, State University of Rio de Janeiro, Rio de Janeiro, Brazil

**Keywords:** Diabetic Nephropathy, Microalbuminuria, Incidence, Risk Factors, Type 1 Diabetes

## Abstract

**Background:**

The presence of microalbuminuria can be associated with overt nephropathy and cardiovascular disease in patients with type 1 diabetes (T1D). We aimed to determine the incidence and evaluate the baseline predictors for the development of microalbuminuria in patients with T1D.

**Methods:**

This study is a longitudinal cohort study of 122 normoalbuminuric patients with T1D who were receiving routine clinical care at baseline. A detailed medical history was taken, and a physical examination was performed at baseline. All of the patients were regularly examined for diabetes-associated complications. An analysis of predictors was performed using the Cox regression.

**Results:**

Over 6.81 (3.59-9.75) years of follow-up, 50 (41%) of the patients developed microalbuminuria. The incidence density was 6.79/100 people per year (95% CI 5.04-8.95), and the microalbuminuria developed after 5.9 (2.44-7.76) and 11 (5-15) years of follow-up and diabetes duration, respectively. After an individual Cox regression, the baseline variables associated with the development of microalbuminuria were age, age at diagnosis, duration of diabetes, systolic and diastolic blood pressure, fasting glycemia, body mass index (BMI), total cholesterol and triglycerides levels, cholesterol/HDL ratio and a family history of type 2 diabetes.After a multivariate Cox regression, the only independent factors associated with the development of microalbuminuria were BMI [HR 1.12 (1.03-1.21)] and cholesterol/HDL ratio [HR 1.32 (1.05-1.67)].

**Conclusions:**

A higher BMI and cholesterol/HDL ratio increased the risk of developing microalbuminuria in young patients with T1D after a short follow-up. Both risk factors are modifiable and should be identified early and followed closely.

## Background

Type 1 diabetes (T1D) leads to an increased risk of morbidity and early mortality due to chronic complications [1] affecting both the micro- and macro-vasculature.

Diabetic nephropathy (DN) is one of the most serious chronic complications of T1D, affecting approximately 20-30% of patients and increasing the risk of cardiovascular disease [2] and end-stage renal disease [3].

Diabetic nephropathy consists of several stages. Early microalbuminuria is considered to be one of its most important predictors [4] and is also associated with the development of cardiovascular disease [5]. Furthermore, the progressive decline of renal function in patients with T1D is an early event that occurs in a large proportion of patients with microalbuminuria [6]. Metabolic and hemodynamic factors contribute to the development of diabetic nephropathy, probably by interacting with a genetic susceptibility [7,8].

The aim of this longitudinal study was to determine the incidence of and predictors for the development of microalbuminuria in a sub-set of Brazilian patients with T1D.

## Methods

We conducted a longitudinal observational study including outpatients with T1D who regularly attended the diabetes clinic at the University Hospital Pedro Ernesto, a tertiary referral hospital of the State University of Rio de Janeiro, Brazil. The inclusion criteria were a T1D diagnosis, regular follow-up at our clinic and agreement to participate by means of informed consent. Exclusion criteria were less than 2 follow-up visits, the presence of either incipient or clinical nephropathy at baseline and end-stage renal disease. The initial cohort started in January 1991 when 110 patients were invited to participate in a study of the complications of diabetes. Initially, 50 patients were enrolled in the protocol, and these data were published elsewhere [9]. A dynamic cohort analysis was then performed. During the follow-up, the patients were seen in our clinic and received routine clinical care.

All of the patients were regularly examined for complications of diabetes. At baseline, detailed medical histories were taken, and physical examinations were performed. The data of interest included gender, age, age at diagnosis of diabetes, first-degree family history of type 2 diabetes (T2D), duration of the disease and daily insulin dose. All of the participants provided three accurately overnight urine samples with an interval of at least one week between collections within a period of six months. The participants were instructed to collect urine under their usual conditions, avoiding intensive physical activity. The urine was collected into containers without preservatives. The urinary volume was recorded, and the aliquots were stored in glass tubes at -20°C until analysis (within 2 months). Urinary albumin concentration was estimated using a solid-phase competitive chemiluminescent enzyme immunoassay (Immulite 1000 Systems, DPC Medlab, Los Angeles, CA, sensitivity of 0.9 μg/ml) with intra-assay and inter-assay coefficients of variation of 2.4% and 5.7%, respectively. The patients with albumin excretion rates (AER) of < 20 μg/min during the follow-up were classified as normoalbuminuric. Microalbuminuria was defined as an AER of ≥ 20 and < 200 μg/min in at least two out of three of the overnight urine specimens collected.

Each urine specimen was tested for the presence of urinary infections and if present, the urine was discarded, and a new sample was collected after treatment. Normal serum creatinine levels (0.8-1.4 mg/dl) and normal urinary sediments (absence of proteins, red blood cells, hemoglobin, white blood cells, nitrites and casts) were used to exclude primary renal disease.

Fasting blood samples were obtained on the same day as the overnight urine delivery. All of the patients were given their usual insulin doses after the fasting blood collection. Blood was collected two hours after the patients' usual breakfast for the assessment of postprandial glycemia.

Triglyceride, total cholesterol, high-density lipoprotein (HDL) cholesterol, fasting plasma glucose (FPG) and plasma creatinine levels were measured by an enzymatic procedure using an auto analyzer (Cobas - Mira Roche). Low-density lipoprotein (LDL) cholesterol levels were calculated using the Friedwald method[10]. Glycated hemoglobin (A 1c) levels were determined by HPLC (Hitachi L-9100) with reference range of 2.6% to 6.2%.

An ophthalmologist examined all of the retinas by fundoscopy through dilated pupils using slit lamp stereoscopic biomicroscopy, and the patients were classified into two groups depending on the presence or absence of either nonproliferative or proliferative retinopathy. The more severely affected eye was used for the evaluation.

Blood pressure was measured 3 times after 5 minutes of rest in the supine position by the same observer using a standard mercury sphygmomanometer. The mean of these values was then calculated. Weight and height were measured to the nearest 0.1 Kg and 0.1 cm, respectively. Body mass index (BMI-Kg/m^2^) was calculated, and the patients were classified into two groups (normal, BMI < 25 kg/m^2 ^for adults and < 85^th ^percentile for children and adolescents or overweight/obese, when higher values were obtained) [11].

The observational design was approved by the local ethics committee.

Numeric values are expressed as medians (interquartile ranges), and categorical variables are expressed as counts (percentages). The analysis was performed using the statistical package Stata version 10 software. Cox regression univariate analysis was performed on each baseline variable. The patients contributed until they developed microalbuminuria, and the outcome was defined as the time elapsed until the development of microalbuminuria. Cox's proportional hazards model was used to estimate the hazard ratios and 95% confidence intervals. The variables were eligible to enter the multivariate model if the p < 0.25 in univariate Cox regression. The proportional hazards assumption was verified by analysis of the Schoenfeld residuals and assessment of a graph of the survival function versus the survival time. The incidence rate of microalbuminuria is expressed as cases/100 patients/year. Receiver operating characteristic (ROC) analysis was performed to identify the threshold value of the baseline AER that predicted the development of microalbuminuria.

## Results

The baseline clinical data for all of the patients in the cohort analysis is presented in Table 1.

**Table 1:** Baseline characteristics of the patients with type 1 diabetes

	Total	Microalbuminuric group	Normoalbuminuric group
**N**	122	50	72

**Gender (Male)**	59(48.4)	21 (42.0)	38 (52,8)

**Age (years)**	17 (12.0-25.2)	20 (14-26)	14 (9-21)

**Age at diagnosis (years)**	12 (7.0-9.0)	14 (9-23)	11 (6-18)

**Duration of diabetes (years)**	3 (1.0-5.6)	4.0 (2.0-8.3)	2.0 (1.0-4.5)

**Insulin dose (IU/Kg/day)**	0.7 (0.46-0.99)	0.73 (0.59-1.12)	0.63 (0.44-0.98)

**Systolic blood pressure (mmHg)**	103 (97-110)	103 (100-110)	104 (97-110)

**Diastolic blood pressure (mmHg)**	68 (60-73)	69 (61-73)	68 (60-73)

**Fasting plasma glycemia (mg/dl)**	150 (110-233)	163 (123-253)	147 (108-224)

**Two-hour post-prandial glycemia (mg/dl)**	221 (148-312)	229 (148-345)	214 (149-306)

**A1c (%)**	7.2 (6.5-8.11)	7.0 (6.5-7.9)	7.3 (6.6-8.5)

**Total cholesterol level (mg/dl)**	158 (140-191)	164 (143-197)	157 (138-183)

**Trygliceride level (mg/dl)**	60 (46-81)	68 (50-97)	55 (44-73)

**HDL cholesterol level (mg/dl)**	49 (41-57)	49 (42-56)	48 (39-63)

**LDL cholesterol level (mg/dl)**	105 (86-137)	113 (92-14)	101 (83-121)

**Cholesterol/HDL ratio**	3.3 (2.7-4.1)	3.4 (2.8-4.2)	3.2 (2.6-4.0)

**LDL/HDL ratio**	2.3 (1.6-3.1)	2.5 (1.8-3.4)	2.0 (1.5-2.9)

**Plasma creatinine level (mg/dl)**	0.65(0.6-0.8)	0.7 (0.6-0.9)	0.6 (0.6-0.8)

**Albumin excretion rate (μg/min)**	7.0 (4.3-10.2)	7.4 (5.1-11.5)	6.8 (4.1-10.0)

**BMI (kg/m**^**2**^**)**	20 (18-22)	20.7 (18.9 22.7)	19.2 (17.1-21)

**Overweight or obese**	22 (18)	8 (16.0)	14 (19.4)

Presence of retinopathy *	6(5)	2 (4.1)	4 (5.6)

Family history of type 2 diabetes**	24(22)	13 (28,3)	11 (16,4)

Continuous variables are presented as median (interquartile range), and categorical variables are presented as n (%).A1c, glycated hemoglobin; BMI, body mass index; HDL, high density lipoprotein; LDL, low density lipoprotein * 2 evaluations are missing ** Data from 8 subjects are missing

Over the 6.81 (3.59-9.75) years of follow-up, 50 (41%) of the 122 normoalbuminuric patients at baseline fulfilled the criteria for microalbuminuria. This yielded an incidence density of 6.79/100 people/year (95% CI, 5.04-8.95).

The follow-up period and the duration of diabetes until the development of microalbuminuria were 5.9 (2.44-7.76) and 11 (5-15) years, respectively. The final AERs were 32.3 (22-50) ụg/min and 7.35 (4.95-11.6) ụg/min for patients who had microalbuminuria and patients who had persistent normoalbuminuric,respectively.

The baseline AER did not significantly predict the development of microalbuminuria in the ROC analysis (AUC = 0.57, 95% CI 0.46-0.67), and there was no single best threshold value.

The baseline variables that were selected to enter the multivariate proportional hazards regression were age, age at diagnosis, duration of diabetes, systolic blood pressure (sBP), diastolic blood pressure (dBP), FPG, BMI, total cholesterol and triglycerides levels, cholesterol/HDL and LDL/HDL ratio and a family history of T2D.(Table 2).

**Table 2:** Cox proportional hazard model of baseline risk factors for development of microalbuminuria in 122 patients with type 1 diabetes who were followed for seven years

Baseline Data	Univariate Analysis Hazard Ratio (95% CI) p value		Multivariate Model Hazard ratio (95% CI) p value	
**Age**	1.03 (1.00-1.06)	0.026		

**Age at diagnosis**	1.03 (0.99-1.06)	0.123		

**Gender**	0.92 (0.52-1.63)	0.779		

**Duration of diabetes**	1.03 (0.98-1.07)	0.247		

**Insulin dose**	1.34 (0.77-2.32)	0.302		

**AER**	1.01 (0.96-1.07)	0.660		

**Systolic blood pressure**	1.02 (0.99-1.04)	0.170		

**Diastolic blood pressure**	1.02 (0.99-1.06)	0.210		

**A1c**	1.02 (0.87-1.20)	0.810		

**Two-hour post-prandial glycemia**	0.99 (0.99-1.00)	0.294		

**Fasting glycemia**	1.00(0.99-1.01)	0.234		

**BMI**	1.12 (1.03-1.22)	0.006	1.12 (1.03-1.21)	0.01

**Total cholesterol level**	1.01 (1.0-1.01)	0.042		

**HDL cholesterol level**	0.99 (0.98-1.02)	0.930		

**Triglyceride level**	1.00 (0.99-1.01)	0.08		

**LDL cholesterol level**	1.01 (1.00-1.01)	0.045		

**Cholesterol/HDL ratio**	1.32 (1.07-1.63)	0.010	1.32 (1.05-1.67)	0.019

**LDL/HDL ratio**	1.26 (0.97-1.62)	0.079		

**Presence of retinopathy**	1.37 (0.33-5.68)	0.667		

**Family history of type 2 diabetes**	2.03 (1.06-3.92)	0.044		

BMI, body mass index; AER, albumin excretion rate; A1c, glycated hemoglobin; HDL, high density lipoprotein; LDL, low density lipoprotein

After multivariate Cox regression, the independent factors that were associated with the development of microalbuminuria were BMI and cholesterol/HDL ratio (Table 2).

## Discussion

In our prospective study of normoalbuminuric patients with juvenile-onset T1D, 41% developed microalbuminuria after an 11-year duration of diabetes and 5.9 (2.44-7.76) years of follow-up, resulting in a greater overall crude incidence and incidence density of microalbuminuria than those reported in other studies [2,12-17].

In addition to the most described predictors of the progression to diabetic nephropathy and AER levels in T1D, such as duration of diabetes [11,18,19], blood pressure [11,18,19] and lipid levels [18-20], the development of microalbuminuria in our population was determined by age, age at diagnosis, BMI, FPG and a family history of T2D.As previously reported[21,22],A1c did not predict the development of microalbuminuria in our population,which is in contrast to other reports[12-14,18,19].

In our study, the levels of AER at baseline also did not predict the development of microalbuminuria, which is in contrast to other studies where higher initial levels of AER predicted the progression to microalbuminuria [13,14].

In addition to the classical risk factors, others may influence the development of microalbuminuria [23]. Considering the increasing frequency of weight gain and obesity in patients with T1D over the last decade, these clinical conditions that result in insulin resistance may be involved in the pathology of microalbuminuria [24]. In our patients, most of who were young adults of a normal weight, the only independent factors associated with the development of microalbuminuria were BMI and the cholesterol/HDL ratio.

Intensive insulin treatment, although beneficial for decreasing the risk of diabetic complications, can result in weight gain [25]. The effects of weight gain may be intensified when not accompanied by good glycemic control. It has been postulated that a first-degree family history of T2D, already reported to be higher in Brazilian patients with T1D compared to non-diabetic patients [26], predisposes these patients to a higher risk of weight gain and insulin resistance.

The features of metabolic syndrome that are associated with insulin resistance, which tend to increase with disease progression [27], were found to be risk factors for overt nephropathy and renal injury [13,14,28-31], due in part to low-grade inflammation and oxidative stress [32]. Patients with T1D can be more susceptible to LDL oxidation *in vitro *[33] and have higher levels of acute-phase proteins compared to non-diabetic subjects [34].

Higher levels of HDL were found to have a protective effect against the development of albuminuria in long-standing type 1 diabetic patients [35]. However, even when they had normal or slightly high plasma HDL cholesterol levels, type 1 diabetic patients were predisposed to the early development of atherosclerosis because of the loss of the ability of HDL to protect arteries from the inhibition of the endothelium-dependent relaxation induced by oxidized-LDL [36]. Total cholesterol [14] and LDL-cholesterol [13,20] levels were also associated with the progression to microalbuminuria.

DN is also associated with cardiovascular disease [37], and dyslipidemia is considered a risk factor for both conditions [38]. In addition, cholesterol levels increase as DN progresses and also correlate positively with the rate of fall in the glomerular filtration rate[39].

It should be noted that we can intervene to mitigate many of these factors. However, the effects of these interventions in affording or delaying the onset of renal injury in these patients must be addressed. Furthermore, microalbuminuria reverts without specific treatment in a significant percentage of patients[40].

These facts are of great importance in deciding the proper time to initiate a specific treatment, particularly because most of the patients are young people of reproductive age.

The limitations of our study include the variable follow-up time and the high number of events. In fact, the final AER levels in those patients who progressed to microalbuminuria were close to the normal range of albuminuria, and this group may have included those patients who spontaneously revert to normoalbuminuria. Family history of hypertension was absent and ethnicity was difficult to characterize in the study population based on ectoscopy or self-report [41], so these data could not be evaluated as risk factors.

## Conclusions

Higher BMIs and cholesterol/HDL ratios at a young age increased the risk of developing microalbuminuria in patients with juvenile-onset T1D after a short follow-up. Both risk factors are modifiable and should be identified early. We suggest that weight and lipid profiles must be followed closely in patients with T1D.

## List of Abbreviations

AER: albumin excretion rate; BMI: body mass index; dBP: diastolic blood pressure; DN: diabetic nephropathy; FPG: fasting plasma glucose; sBP: systolic blood pressure; T1D: type 1 diabetes; T2D: type 2 diabetes

## Competing interests

The authors declare that they have no competing interests.

## Authors' contributions

RAC participated in the design of the study, data collection, drafted the manuscript.PCBS participated in the collection of data and helped to draft the manuscript.RN conducted ophthalmologic evaluations, participated in the study design and helped to draft the manuscript. BS participated in the design of the study and performed statistical analysis.MBG participated in the design and coordination, helped to draft the manuscript and gave final approval.All authors read and approved the final manuscript.
